# Editorial: Recent Advances on Grapevine-Microbe Interactions: From Signal Perception to Resistance Response

**DOI:** 10.3389/fpls.2020.01164

**Published:** 2020-07-29

**Authors:** Aziz Aziz, Michele Perazzolli, David Gramaje, Eva Maria Zyprian, Dario Cantu

**Affiliations:** ^1^ Induced Resistance and Plant Bioprotection (RIBP), SFR Condorcet FR-CNRS 3417, University of Reims, Reims, France; ^2^ Center Agriculture Food Environment (C3A), University of Trento, San Michele all’Adige, Italy; ^3^ Department of Sustainable Agro-Ecosystems and Bioresources, Research and Innovation Centre, Fondazione Edmund Mach, San Michele all’Adige, Italy; ^4^ Instituto de Ciencias de la Vid y del Vino (ICVV), Consejo Superior de Investigaciones Científicas, Gobierno de La Rioja, Universidad de La Rioja, Logroño, Spain; ^5^ Institute for Grapevine Breeding Geilweilerhof, Julius‐Kuehn Institute, Siebeldingen, Germany; ^6^ Department of Viticulture and Enology, University of California Davis, Davis, CA, United States

**Keywords:** disease management, induced resistance, breeding, biocontrol, plant immunity, plant microbiome, beneficial microbes, *suppress Vitis vinifera* L

Grapevine (*Vitis vinifera* L.) is one of the most economically important cultivated perennial plants worldwide and is susceptible to an array of diseases caused by pathogenic fungi, bacteria, viruses, and oomycetes. In recent decades, extensive efforts have been made to reduce the use of chemical pesticides in viticulture by developing and deploying more sustainable alternative solutions. This Research Topic covers the most recent advances on both basic and applied research on grapevine-microbe interactions. It provides an overview on (i) the different resistance loci/genes evolved in grapevine species and their application for breeding new resistant cultivars; (ii) the molecular basis of grapevine susceptibility and resistance to pathogens; (iii) the biocontrol and integrated grapevine disease management including induced plant innate immunity by beneficial microbes and natural elicitors; and (iv) the functional properties of endophytic or rhizospheric microbial communities that promote plant health.

Breeding for resistant genotypes represents a promising alternative to control powdery mildew (*Erysiphe necator*) and downy mildew (*Plasmopara viticola*) in grapevine. The dissection of the different resistance loci/genes evolved in grapevine species might provide information on the mechanisms involved in resistance. In this regard, using quantitative trait locus (QTL) mapping Vezzulli et al. have investigated metabolic homeostasis related to some loci, as a means to better improve grapevine resistance. They reported the identification of 46 novel metabolic QTLs linked to 30 phenolics-related compounds. They further analyzed the expression of 11 genes in genotypes resistant to *P. viticola* (*Rpv3-3*+/−) and revealed that the *Rpv3-3* haplotype is associated with stilbenoid induction. The evaluation of genetic resistance of vegetative organs is traditionally based on measuring pathogen growth and/or disease symptoms on leaf discs. Bove et al. described another method based on the measurement of components of resistance (RCs) taking into account the infection frequency, duration of latent period, lesion size, production of sporangia, infectious period, and infectivity of sporangia produced. The RC scores were compared with the visual score proposed in the 2nd Edition of the International Organization of Vine and Wine (OIV). According to the authors, the measurement of the RCs and their incorporation into a model that simulates their effect on downy mildew development, would be a more accurate method for phenotyping resistance level. A review article by Pirrello et al. presented an overview on studies that focused on the genetic variability of grapevine pathogens, especially of the causal agents of powdery mildew, black rot and anthracnose. They explored various aspects including disease symptom assessment, nonsynthetic chemicals, and organic control measures. They also highlighted the usage and development of molecular markers and barcoding, coupled with genome sequencing, to better understand genetic variability and resistance mechanisms of these pathogens to fungicides, and to identify candidate effectors involved in the pathogenicity and host resistance mechanisms.

An important aspect dealing with the energy costs of pathogen infection and inducible defenses in terms of carbon production and utilization by the host was also addressed in this Research Topic. Four original research papers provided new insights into aspects of biological function of sugar partitioning and photosynthesis by examining different pathosystems. Based on different cultivars with contrasting phenotypes, Cardot et al. highlighted that the availability of sugar resources for either the host or the fungus is crucial for the outcome of the interaction between grapevine and *Eutypa lata*, the causal agent of eutypa dieback. Membrane transporters, like SWEET (Sugar Will be Eventually Exported Transporters) transporters, are the targets of extracellular pathogens and seem to be decisive in the control of the competition for sugar. Meteier et al. showed that the overexpression of *SWEET4* in grapevine hairy roots leads to improved resistance to *Pythium irregulare*, a soilborne necrotrophic pathogen. This work highlights the key role of sugar transport mediated by SWEET transporters for secondary metabolism regulation and pathogen resistance in grapevine. Breia et al. reported on the importance of SWEET transporters, especially *SWEET7* in sugar mobilization during grape berry development and in response to *Botrytis cinerea* infection. Nogueira Junior et al. evaluated the photosynthetic cost of the activation of leaf defense responses in a downy mildew resistant cultivar. The authors showed that the defense response against *P. viticola* causes a photosynthetic cost to grapevines, which is not reversible even 12 days after the pathogen infection.

Grapevine is also colonized by a wide variety of beneficial rhizospheric and endophytic microbes, which can promote plant growth and health. Some of these microbes can act as biological control agents, thanks to their ability to prime plant immunity, known as induced systemic resistance (ISR), and to their antagonism against pathogens. Like in pathogens, microbe-associated molecular patterns (MAMPs) are associated with beneficial microorganisms. The perception of MAMPs triggers the activation of the plant’s immune response. The recognition of these natural elicitors and their function in grapevine immunity were reviewed by Héloir et al. The authors described in detail the chemical nature, the structural diversity, and how MAMPs, as well as host-derived damage-associated molecular patterns (DAMPs), are perceived by grapevine cells and subsequently activate grapevine immunity, resulting in some cases in disease resistance. Using cultivars with different levels of susceptibility to downy mildew, Lakkis et al. showed that the endophytic bacterium *Pseudomonas fluorescens* PTA-CT2 affects hormonal status and enhances photosynthetic efficiency in both susceptible and partially resistant cultivars. The beneficial bacterium also induces ISR against *P. viticola* and *B. cinerea* by priming common and distinct defensive pathways, depending on the basal resistance of the cultivar and the pathogen lifestyle. The biocontrol activity of *Bacillus subtilis* PTA-271 was demonstrated by Trotel-Aziz et al. against Botryosphaeria dieback, caused by the fungus *Neofusicoccum parvum*. The authors reported that the main toxins (-)-terremutin and (R)-mellein produced by *N. parvum* may suppress grapevine immunity to promote Botryosphaeria dieback symptoms. Interestingly, the beneficial bacterium *B. subtilis* PTA-271 not only antagonizes the pathogen, but also primes host immune response and detoxifies both fungal phytotoxins. Cobos et al. characterized the virulence activity of necrosis and ethylene inducing peptide 1 (NEP1)-like proteins of another Botryosphaeria dieback pathogen, namely *Diplodia seriata*. By analyzing the expression of the NEP encoding genes in conditions mimicking plant infection and expressing two of the four identified NEPs as recombinant proteins, the authors highlighted differential necrotic and cytolytic activity of NEP1-like proteins under *in vitro* and greenhouse conditions.

Other contributions to this Research Topic focused on the endophytic and rhizospheric microbiome with respect to their functional and beneficial effect on plant fitness and health. Niem et al. characterized the beneficial effect of endophytic bacteria isolated from grapevine wood to control trunk diseases. The authors identified *Pseudomonas* spp. as dominant taxon among the bacterial community in canker-free grapevine tissues, indicating their ability to colonize and play a role in grapevine health. Further tests revealed antagonistic activity of distinct fluorescent Pseudomonas isolates against Esca, Botryosphaeria and Eutypa dieback, making them potential candidates to control trunk diseases. In their review, Pacifico et al. summarized the current understanding of the role of endophytes in grapevine fitness and tolerance to biotic and abiotic stresses. The authors explored different mechanisms related to colonization and the interaction of endophytes with plant metabolism, as well as their efficiency to confer plant fitness and disease resistance. The authors also highlighted the role of the environment and discussed strategies of endophyte application for a more sustainable viticulture. Other works used amplicon metagenomic approaches to explore the plant microbiome, in order to understand the diverse structure and functions in plant fitness and health. Berlanas et al. evaluated the effects of rootstock genotype on bacterial and fungal communities in the rhizosphere, and found that microbial composition and diversity were significantly driven by the rootstock type in mature vineyards. Similarly, Del Frari et al. characterized the wood fungal composition in different areas of the stem and canes, and assessed the link between mycobiome and leaf symptoms of Esca disease. Although the spatial analysis revealed differences in diversity, taxa abundances and in tissue specificity, foliar symptoms were not directly linked with the fungal community in the wood. In another report, Deyett and Rolshausen performed a comprehensive culture-independent microbiome analysis from the sap of grapevine in the context of Pierce’s disease caused by *Xylella fastidiosa*. The authors identified a core microbiome of the sap composed of bacterial and fungal taxa with antimicrobial and plant growth promoting capabilities that were present throughout the growing season, while microbial community profiles were influenced by disease condition and plant phenology. In a second contribution, Del Frari et al. dissected how fungicides commonly applied against downy and powdery mildew agents affect the wood mycobiome, including wood pathogens such as *Phaeomoniella chlamydospora* under greenhouse conditions. The authors suggested that the resident fungi that colonize the endosphere of grapevines are affected by different fungicides, some of which would be effective against *P. chlamydospora*, as well as the early colonization success of a consortium of fungal endophytes. Lastly, Gan et al. provided a valuable contribution to the field of microbiota associated with the grapevine crown gall disease caused by *Agrobacterium* spp. or *Allorhizobium* spp. in seven infected vineyards with separate geographic distributions. Using 16S amplicon sequencing the authors showed that the crown gall microbial community varies significantly across sampling sites and/or climate conditions, and harbors a core microbiome containing *A. vitis*.

Overall, this Research Topic reports on recent advances in grapevine-microbe interactions in both fundamental and applied terms. The papers compiled revealed a growing interest for sustainable solutions for grapevine disease control. This includes the breeding/phenotyping programs towards aerial and trunk diseases, biocontrol strategies and priming plant immunity, the physiological cost linked to plant defense, as well as the exploration of the functional properties of the plant microbiome for a sustainable modern viticulture.

## Author Contributions

All authors listed have made substantial, direct, and intellectual contribution to the work and approved it for publication.

## Conflict of Interest

The authors declare that the research was conducted in the absence of any commercial or financial relationships that could be construed as a potential conflict of interest.

